# Taqsīm as a Creative Musical Process in Arabic Music

**DOI:** 10.3389/fpsyg.2021.640409

**Published:** 2021-06-11

**Authors:** Zaher Alkaei, Mats B. Küssner

**Affiliations:** Department of Musicology and Media Studies, Humboldt-Universität zu Berlin, Berlin, Germany

**Keywords:** taqsīm, Arabic music, musical creativity, composition, improvisation

## Abstract

Creativity plays a major role in various musical contexts including composition, performance and education. Although numerous studies have revealed how creativity is involved in processes of listening, improvising and composing, relatively little is known about the particularities of transcultural creative processes in music. In this article, we aim to shed light on the creative musical processes underlying taqsīm performance in Arabic music. To that end, qualitative interviews have been conducted with three Berlin-based oud players from Syria. Results of a thematic content analysis show that taqsīm encompasses multiple components (e.g., a flexible form and dependency on maqam as well as tonal music) and serves various functions such as developing artistic individuality. Moreover, taqsīm is affected by interactions between tradition and novelty. We discuss the interview data within the cross-cultural experiential model of musical creativity developed by Hill (2018), offering a fresh approach to studying taqsīm which goes beyond established concepts such as the improvisation-composition continuum.

## Introduction

The relationship between music and creativity has been studied from numerous angles, including creativity and listening (Peterson, 2006), creativity in educational settings (Crow, 2008; Odena and Welch, 2012), and creativity and musical performance (Deliège and Wiggins, 2006). Schiavio and Benedek (2020) have recently argued that previously posed theories of creativity in musical and non-musical domains distinguished between the following four types of creativity: (1) individual creativity, which focuses on comparing the creative products of individual subjects and linking them to individual differences (Auh, 1997; Villarreal et al., 2013); (2) collective creativity (Clarke and Doffman(eds), 2017; Bishop, 2018), which is focused on distributed activity across members of music ensembles as well as on composer-performer creative collaboration. This approach stresses the social aspect of music-making. Researchers like Burnard (2012a) have argued that a singular understanding of musical creativity (like the one achieved by individual composers) resulted in a limited definition of creativity. Alternatively, a collectivist perspective on music can be a source of meaningful socio-historical representations of objects and actions. From such a viewpoint, the audience (as a community) plays a crucial role in influencing and co-creating creativity. Further, concepts of creativity can be rooted within socio-cultural contexts, where creativity practices can be conceptualized as being situated and collective (Kenny, 2014). The social and cultural contexts in which creativity in music occurs allow researchers to investigate a wide scope of musical genres like improvised music (Burnard, 2012a). Psychologists and cognitive scientists are interested in (3) domain-general perspectives on creativity (Beaty et al., 2013), arguing that domain-general abilities influence and may predict domain-specific abilities. A contrary approach is to focus on (4) domain-specific perspectives on creativity (Baer, 2015). In such studies, it is argued that creativity in one domain does not predict creativity in other unrelated domains. Schiavio and Benedek (2020) highlight that these aspects should not be understood as alternatives. To reduce the tension between these poles, they draw from a range of scholarship in music and enactive cognitive science, and suggest that creative cognition may be best understood as a process of skillful organism-environment adaptation that can be cultivated continually. Moreover, other researchers explored connections and tensions between creativity research and developmental psychology (Sawyer et al., 2003). In addition to that, musical creativity can be studied with the paradigm of embodied cognition (Nagy, 2016; Van der Schyff et al., 2018).

With regard to musical activities, Hargreaves et al. (2011) have argued that there are common mental structures underlying the three main activities of music: invention (composing and improvising), performance of music, and listening to it, and that these structures reveal imagination and creativity (see also Hargreaves, 2012). The activities of music invention (i.e., composing and improvising) are often presented in the literature as endpoints of a continuum (Pressing, 1984; Sarath, 1996; Lehmann, 2005; Goldman, 2019), although a strict separation can be doubted from a psychological perspective (Lehmann and Kopiez, 2010). It is also questionable whether cultural constructs such as “composition” and “improvisation” can be adapted to examine the wide range of *transcultural* creative processes in different musics. We use the term transcultural to denote that (musical-) cultures may have boundaries, yet these boundaries are not absolute or even clearly defined. Thus, a transcultural perspective indicates “a procedural act of a cultural overstepping of boundaries or the condition which results from this overstepping of boundaries” (Kim, 2017, p. 20). Moreover, creative thinking in music is not restricted to one genre, style or historical era, rather it is a general feature found in many musical activities (Campbell, 1990). Studying musical creativity would therefore benefit from including a wider range of musical practices and their components.

Besides studying music practices from a transcultural point of view, the mobility of musical genres and practices as well as studying migrants’ music may reveal new interconnections between creative musical processes. Baily and Collyer (2006) point out that the cultural and spatial distance between the cultural background of the migrants and the culture of the settlement place affect the musical practice in the migration situation. Sorce Keller and Barwick (2012) note that studying musical attitudes, tastes, and practices can provide valuable insights into the details of processes like assimilation, co-habitation and the maintenance of distinctive cultural traits. Furthermore, Baily and Collyer (2006) state that cultural innovation and enrichment may result from migration.

Here, we propose that shedding light on taqsīm—a musical practice in Arabic, Turkish and Persian cultures—can enrich our perspective on creative processes in music and inform the wider musicological and cognitive science discourse. Taqsīm is a musical practice dependent on oral transmission, which may allow space for novelty but may make it vulnerable to significant changes in a globalized world. It also has an open and more flexible form compared to other Arabic compositional forms or European forms of classical music. The term taqsīm (plural: taqāsīm) refers to a non-metric improvised solo form in Arabic music. The word itself means “division,” implying variation and improvisation (Nettl, 1974). Marcus (1993) points out that taqāsīm are not simply free-formed products of the musician’s fantasy. He notes that the player improvises according to a complex set of pre-established rules and conventions. Taqsīm, according to Marcus (1993), is a highly valued musical genre in Arabic music because it gives the players the opportunity to present their own creations instead of relying on the compositions of others. Racy (2000) elaborates on this point by adding that the ability to play taqsīm is generally considered a characteristic of good musicianship in the Arab world. Racy also notes that playing taqsīm, which may be heard in combination with non-improvised compositions or alone, is known to require extraordinary skill, talent, and inspiration. Watson (2012) notes that taqsīm can be performed unaccompanied or accompanied. In accompanied settings, taqsīm is often played over a single-pitch drone without rhythmic accompaniment. Alternatively, taqsīm can also occur over a repeated ostinato, or within a repeated percussion cycle.

### Composition and Improvisation

Taqsīm is often seen as an improvisatory genre (Touma, 1971; Nettl and Riddle, 1973; Racy, 2000; Shannon, 2003; Watson, 2012). Yet, a strict separation between composition and improvisation can be doubted. Nettl (1974) highlights that improvisation has been considered as a type of composition that characterizes musical cultures without notation. This type of composition depends on releasing sudden impulses to music through direct production of sound. According to Nettl, it has been argued that improvisation ends where notation begins, while others contend that certain non-western cultures, which do not use notation, distinguish between the two processes by internally classifying their musics. Taqsīm is rarely notated—maybe only for theoretical analysis by musicologists—yet, as Nettl notes, the absence of notation is not enough to describe some musical genres as improvisation. Improvisation and composition can be seen as creative music-making processes (Lehmann and Kopiez, 2002). Moreover, Nettl (1974) proposed a continuum-based definition where composition and improvisation are thought of as being at the “opposite ends of a continuum.” However, it is not clear whether it is sensible to locate taqsīm on such a linear binary continuum, because we would still need to define improvisation and composition to establish such a continuum. Despite Nettl’s suggestion to consider composition and improvisation as being at the opposite ends of a continuum, the wider observation of many musical traditions around the world led him to conclude that “perhaps we must abandon the idea of improvisation as a process separate from composition and adopt the view that all performers improvise to some extent” (Nettl, 1974, p. 19). He later expresses further doubt, stating that “we probably should never have started calling it improvisation” (Solis and Nettl, 2009, ix). Similarly, Nooshin (2017) suggests that the continuum model did not go far enough. Nooshin argues that a better understanding of creative processes would ultimately require a complete dissolution of the composition/improvisation dichotomy. Moreover, Lehmann and Kopiez (2002) point out that composition and improvisation are much more akin than commonly assumed. By analyzing optimization processes and constraints on the creative performer and process, Lehmann and Kopiez show that both processes tap into the same mental mechanisms and require similar prerequisites. They conclude that such a view opens up new ways of investigating generative creativity in music.

However, some researchers may argue that there are important differences between composition and improvisation. Sloboda (1986) argues that what distinguishes improvisation from composition is the pre-existence of a large set of formal constraints which comprise a “blueprint” or “skeleton” for the improvisation. Sloboda notes that the improviser, who depends on a “blueprint,” can manage without much of the composer’s usual decision-making process especially in aspects like structure and direction. Sloboda argues that while the composer may keep rejecting possible solutions during composing until finding a more suitable one, the improviser has to do with the first solution that comes to mind. Moreover, temporality is another aspect which may distinguish composition and improvisation. Sarath (1996) argues that while the improviser experiences time in an “inner-directed” manner, where the present is so central in comparison to the past and future, the composer has an “expanding” temporality, which means that the “temporal projections may be conceived from any moment in a work to past and future time coordinates” (Sarath, 1996, p. 1).

Moreover, Mazzola et al. (2011) give a detailed characterization of the creativity processes in composition and improvisation. The creativity process in composition can be characterized as: (1) reliance on symbolic musical objects as elements of a semiotic context which unfolds in a logical time (the rules that govern the shape and arrangement of material in the parameter space of the symbolic events); (2) reliance on basic semiotic architecture which manipulates symbols in a referential network that operates in independence of material time; and (3) a separation of material components from the transformation rules. The creativity process in improvisation can be characterized as: (1) reliance on a system of gesture that does not allow for abstract symbols; (2) reliance on managing of gestures through the connectivity of gestures within hypergestures; and (3) no separation of components from the transformation rules.

However, Mazzola et al. (2011) add that both composition and improvisation can be seen from their generative aspect, thus one can say that “improvisation is instant composition while composition is slow-motion improvisation” (2011, p. 245). In this sense “[t]he generative forces of the compositional logic are not necessarily logical, but they may very well be nourished by gestures of memory, dreams, and yearnings.” (2011, p. 246). Moreover, “[t]he imaginary space-time of improvisation is in itself a kernel structure for a compositional approach to improvisation since it creates a space for musical construction as if we were working out a compositional preconception.” (2011, p. 247).

Rose et al. (2012) also challenged the differentiation of improvisation and composition by conceptualizing improvisation as an instant composition. They add that “[r]eal-time composition becomes realized by means of the body and embodied knowledge and the body can be thought of as the site of improvisation.” (2012, p. 210).

As the literature discussed so far suggests, it is not adequate to assume that taqsīm is simply an improvisatory genre. Instead, it could be more revealing to examine taqsīm as a broader creative music-making process. Glãveanu (2019) proposes three different types of logic that can be used to study creativity culturally: (1) the logic of comparison, which focuses on comparability of creativity-related phenomena and is used in most cross-cultural research; (2) the logic of exploration, which highlights differences in creativity between individuals and groups, mostly used in cultural or sociocultural research; and (3) the logic of understanding, which is used in the research depending on local and bottom-up definitions and categories.

Burnard (2012a) provides a rich framework to study musical creativity. She argues that musical creativities can take many forms, may have many functions, and are contained in personal and sociocultural contexts. She distinguishes between forms of authorship, mediating modalities and practice principles of different musical practices. Moreover, according to her, these elements mediate between individual, social and cultural systems. Yet, she distinguishes between composed music and improvised music – a distinction which can be doubted, as argued above.

### Hill’s Experiential^1^ Model of Musical Creativity

Another framework in which the underlying processes of taqsīm may be discussed more fruitfully is provided by Hill (2018). According to Hill, creativity can be seen as a fundamental human desire and activity that is culturally embedded and socially regulated. She conducted in-depth qualitative research with more than one hundred musicians from different communities. Her research dealing with examples from classical, jazz, and traditional musicians in three cities—Cape Town, Helsinki, and Los Angeles—explored musicians’ thoughts on, and experiences of, the development and practice of musical creativity.

While Hill values the view of the diversity of creative activities and of musical cultures in which they occur as articulated by Burnard’s (2012b), Hill aims to “identify the most common components of creative experience” (Hill, 2018, p. 3). Based on her comparative research in these diverse cultural contexts, she proposes a model of creativity that contains components of generativity, agency, interaction, non-conformity, recycling, and flow. She notes that all these six components were reported across all the musical idioms and cultures in her study (Hill, 2018).

Hill (2018) notes that some cultures may place greater value on some of these components than on others. She notes that the restriction of any one of these components may result in an inhibition of creativity. On the other hand, she argues, supporting all six components can enhance creative potential. Hill (2018) notes that more research is needed to test whether her model is applicable across a broader range of musical cultures.

By exploring three musicians’ reflections on their experience of taqsīm, this study aims to examine whether taqsīm and the musical processes underpinning taqsīm performance in Arabic music may be described within Hill’s model of cross-cultural musical creativity.

## Materials and Methods

### Participants

Participants were three male oud players from Syria: Ala’a, Wassim and Nabil – aged 28, 34, and 37, respectively – who have lived in Berlin since 2015/16. The participants had had private music lessons since childhood and preadolescence. Wassim and Nabil started taking oud lessons when they were 10 years old, while Ala’a began at the age of 13 years. All three are familiar with solfège and musical notation. They used to give public concerts in Syria and expanded their musical activities in Berlin through workshops, solo performances, and participation in ensembles of various sizes. Each participant signed a consent form, and the study was carried out in accordance with the Declaration of Helsinki.

### Materials and Procedure

Half-open questions were used to interview all three musicians. The aim was to explore the musicians’ reflections on their experience of taqsīm. We asked the musicians about the definition, form, function, learning of taqsīm, and also about playing taqsīm in different situations (solo vs. ensemble, with vs. without an audience), as well as about the development of their own taqsīm in the last few years. To what extent is taqsīm free or limited, and how does it compare to composition or improvisation in general? The interviews were conducted in Arabic – the mother tongue of the three participants and the first author – in January and February 2019. The interview with Ala’a took place at the interviewer’s home, Nabil’s at his home, and Wassim’s in the Department of Musicology and Media Studies at Humboldt-Universität zu Berlin. Each interview lasted roughly 35 min. The interview was recorded with a Zoom H5 Audio Recorder and later transcribed into Arabic and then translated into English.

### Data Analysis

A thematic content analysis with the deductive (or top-down) approach was used to analyze the interviews (Hayes, 1997; Boyatzis, 1998; Braun and Clarke, 2006). Reading the original transcription and the translation of the interviews multiple times was the first step in the analysis to get familiar with the data. The translations were checked back with the original recordings to assure accuracy. The literature review on taqsīm gave rise to preliminary codes that were assigned to the data to describe the content. The literature review helped us to code the participant answers by providing us with keywords and central concepts to look for. Examples of such keywords and concepts were: musical environment, maqam music, taqsīm in concerts, interaction with the audience and other musicians, musical material, building blocks, development of taqsīm. Data reduction and inferences about the codes’ meaning were made, and we examined how the themes support the overarching theoretical perspective. Each interview was analyzed separately, later the themes and codes across the three interviews were compared. This enabled us to find similarities and differences in our participants’ answers. Afterward, themes were reviewed and precisely named. A final list of codes and three main themes was produced. These final codes were mapped to the components of Hill’s model. We further discuss the extent to which Hill’s components of creative experience can be found in taqsīm.

## Findings

After conducting the thematic content analysis, the following final codes emerged, which we grouped into the following themes: Interaction – Creation – Freedom/limitation.

Summary of the final codes:

• Instant composition
• Flexible form
• Highlighting the technical skills of the player
• As an introduction for a piece
• A space for interaction
• Learning by imitation
• Creating one’s own taqsīm
• Recycled musical material
• Audience effect on taqsīm
• Playing with an ensemble affects taqsīm
• A meditative state
• Integrating elements from different styles
• Microtonal intervals and other instruments
• Leaving Syria/being in Berlin

A flexible form as the main feature of taqsīm was evident in taqsīm definitions given by all three participants. For Ala’a, besides being “a genre or musical form in oriental music in general and in Arabic music in particular” taqsīm is “a free form that is based on the character of the musician, his repertoire, his cultural heritage of musical phrases, or learned techniques.” Ala’a noted that taqsīm consists of four parts: an introduction, the body of the taqsīm, a kind of development, and a conclusion. Wassim gave a general description of his taqsīm form and stated:

“I build melodies on rhythmic patterns and follow the rules of tetrachords and their order so that I can build musical phrases and develop them and then move on to new musical phrases. Later I set a conclusion, which is a kind of summary of the previous phrases.”

Nabil stated that he would not use a form to structure his taqsīm and added: “taqsīm should be as free as possible.” The answers of the participants highlight that maqam is central to taqsīm. For Ala’a, taqsīm aims to present the maqam of the taqsīm. For Wassim, the taqsīm follows the logic of the maqam. Nabil noted that “the taqsīm introduces the maqam [which will be] sung by the singer.”

There were mixed answers concerning the freedom of taqsīm. The participants pointed out that taqsīm is free but may be limited by other factors such as the setting (many musicians playing together makes it hard to coordinate who will play next and for how long) and time factors (many musicians playing taqsīm may result in a very long performance). However, Wassim noted that taqsīm may be stylistically restricted: “taqsīm follows the tonal rules.” Ala’a and Nabil noted that playing with other bands (especially orchestras or bands that play music with harmonic progression) may limit their taqsīm freedom. These answers support the idea that taqsīm has rules and is not completely free.

When asked about how they learned taqsīm, the participants highlighted multiple learning processes: Ala’a learned taqsīm through listening and being more experienced with Arabic musical tradition. Nabil stated that he always tended to vary the music he learned to play; later, he started to imitate oud masters’ taqsīm. While Ala’a’s and Nabil’s learning processes of taqsīm can be described as autodidactic, Wassim stated that taqsīm was organized by his teachers, with the goal of playing musical elements on the oud.

The three participants accumulate experiences and use their repertoire on the spot of performance. Nabil stated that after playing in many concerts, he has developed many musical ideas he can use in his taqsīm. Besides these experiences in concerts, he develops taqsīm when he is at home practicing. Wassim pointed out two sources for his experience with taqsīm: (1) the interaction with his teachers and (2) experimenting with his instruments. Ala’a depends on his repertoire, which has been built up and collected over several years. The participants highlighted that the musical material played during taqsīm comes from songs, the traditional musical repertoire and the individual experimentation with the instrument and the maqams. These processes indicate that the players internalized stylistic rules that characterize taqsīm by making themselves more familiar with the material through listening and imitating. Yet these rules were not absolute: All players reported influences on their taqsīm music from other musical cultures: For Ala’a, Turkish music, blues and jazz. For Nabil, electronic music and European classical music. For Wassim, music from Azerbaijan and European classical music.

Concerning the role of the audience, the participants’ answers revealed that the interaction with the audience and concert settings have a great influence on taqsīm. Wassim noted that taqsīm functions as an interaction space with the audience, the music itself, and with other players. Yet having an audience may put the player under pressure according to Nabil. Ala’a noted that when he plays in front of an audience there is more enthusiasm. He added that there is a competition between the players to impress the audience.

Moving to Berlin from Syria was seen by our participants as an opportunity to develop their own taqsīm. Ala’a stated:

“The audience I had in Syria was very different from the audience I have in Germany and in Europe in general. I can say that I didn’t really have an audience in Syria because I was giving concerts in small places and people in general were not fans of this style and I always used to cut something from the taqsīm.”

Nabil stated:

“I don’t play taqsīm in Germany the way I play it in Syria. The difference is very big, because here I started to hear jazz improvisations, rock music and live electronic music in a very different way than I heard them in Syria. In Syria, I heard them as recordings and these recordings are pieces of music for me because they are recorded. When I went to hear them again, the music was the same. But when I heard and met the musicians who improvise this music, I started to hear this music differently. Even when the same player improvises again, he repeats the improvisation differently. I started to see them as improvisations and adopting many ideas from them. I have the impression that the ear of the audience [in Europe] is different, so my music changes according to the expectations of the listener. My music has been influenced in this way a lot, not only my improvisation, [but] my compositions also. They got influenced by the music I hear here and by the audience I play for.”

Wassim stated:

“I can say that almost five years after leaving Syria, I have the possibility to really check what I have. Besides the freedom of dealing with a completely new audience, whether a Turkish audience or a European audience. These gave me some freedom. That is, I don’t have to do anything. Everything I do, I want to do and not because I have to do it.”

The answers of our participants indicate that room for individual artistic expression is a primary function of taqsīm. This was evident in the taqsīm definition given by Ala’a: “a free form that is based on the character of the musician, his repertoire, his cultural heritage of musical phrases, or learned techniques.” For Nabil, taqsīm functions as a space in which the player presents his instrument, technique, and musical abilities. For Wassim, taqsīm functions as a space to show the technical skills of the player.

## Discussion

The participants’ answers show that taqsīm has several special characteristics: a musical genre with a flexible form; the importance of maqam to taqsīm; performing taqsīm is governed by rules and not completely free, although such rules are not always explicit and clear to the players themselves; the existence of such rules does not prevent the players from being open to new musical materials; interaction with the audience plays an important role while performing taqsīm; artistic self-expression is central to taqsīm. Seeing taqsīm as an improvisatory genre is not a satisfactory approach, as discussed in the introduction. Thus, we seek to interpret our data within an alternative approach. We will examine whether our data on taqsīm fit within Hill’s model of creativity, paying attention to each component and whether we can find counterparts of these components in our participants’ answers.

### Generativity

According to Hill (2018), generativity is the most basic aspect of the creative process. Hill notes that this aspect can be described as “building,” “making,” “creating,” or “constructing” something. Hill adds that musicians and music scholars mostly agree that composing, improvising, and arranging music are creative generative acts. However, she notes that there is less agreement on whether performing a pre-existing work should be considered creative. A similar position was expressed by Nettl (1974) who suggested that it is self-evident that improvised music requires a greater creative effort on the part of the performer than does composed music. Nettl noted that improvisation may be defined by measuring the degree to which the performer is creatively involved.

In this study, when the participants talked about taqsīm they used expressions denoting the generative nature of the process. Ala’a used verbs like “create” and “build” to talk about taqsīm. Ala’a noted that taqsīm is a creation that uses building blocks from the player’s repertoire. For him these building blocks are:

“the player’s repertoire that he builds and collects over several years. If you are a player in the first level, that is, in your first or second year, you can create a taqsīm, but it can [only] be [either] traditional or a mixture of the songs of Fairuz and Umm Kulthum that you play in your first phase.”

This repertoire and cultural heritage of the musician (like maqams) and the musician’s technical skills play an important role in building taqsīm:

“It is a kind of free form that depends on the musician’s character, his repertoire, his cultural heritage of musical phrases, or learned techniques. It is related to his ability to implement his repertoire in a form determined by rules—to present his feelings through the use of the repertoire he possesses or the technique he has learned, and to present the maqam on which the musician builds his taqsīm, to the listener.”

Nabil also used the verbs “create” and “build” to talk about his taqsīm, with a focus on the idea that with time he started to create his own building blocks, instead of borrowing from other musicians:

“With time and with repetition, I was moving away from the basic musical ideas and the main melodies of the [original] musicians. I started to create a line [melody] that I liked and I [started to] focus on the ideas that I want and like. Like that and with time I became able to create a taqsīm that convinced me.”

Wassim used the verb “build” with melodies and musical phrases. For Wassim, the process is comparable to composition, especially when he speaks about building and developing musical phrases which leads to growth:

“taqsīm from the point of view of classical oriental music […] is tonal music accomplished through building melodies on a rhythmic pattern and they follow the rules of tetrachords. Building phrases and developing them and then move on to new phrases. And later adding a conclusion, which is a kind of summary of the previous phrases.”

Indeed, the connection to composition becomes clear in the following quote from Wassim, even though it is a kind of instant composition:

“What is special about taqsīm is that it is a work that is composed immediately [while playing].”

This generativity is goal-oriented, as mentioned above. Such goals can be summarized as: (1) room for individual artistic expression, (2) interaction with the audience, and (3) support and introduction of pre-composed pieces.

### Agency

The second component of creativity according to Hill (2018) is agency, which was also the most important component for the majority of musicians she interviewed. Agency can be seen as “individuals’ ‘room for action’, and the extent to which we are either subdued by the larger mechanisms of society or can freely decide our ways of being and acting within them” (Karlsen, 2011, p. 110). Such an understanding of agency emphasizes the ability to make one’s own decisions determining musical act and meaning. According to Marcus (1993) taqsīm allows the musicians to demonstrate their abilities as composers and their mastery of their instruments.

Our participants’ answers highlight the importance of agency. For Ala’a, taqsīm is a musical space which is built from “the musician’s character, his repertoire, his cultural heritage of musical phrases, or learned techniques.” This shows that taqsīm style is related strongly to the musician’s personality and individuality. Moreover, playing taqsīm before or within a pre-composed piece of music “opens up a way for any musician to add some of his spirit to the piece and make the piece a combination of several spirits ….” In this way, the player is not only a performer of a pre-composed piece, but rather a co-creator. The player may add, invent or change the form of a piece by playing a taqsīm of his own, before or during the piece, where he has the chance to introduce his individuality as a creator.

Nabil expressed similar ideas. For him, taqsīm is “the most important opportunity for the musician to present himself. He presents himself as a musician with spirit and technique and he reflects the atmosphere of the concert.” Moreover, taqsīm gives the player control over the material of the concert because it is not pre-composed. Thus, the player has the choice to adapt his taqsīm to a different concert situation accordingly.

Wassim stated that taqsīm “should emphasize the player’s technical skills, the understanding of his instrument and interaction with the audience, with the music itself, and with the other players.” Besides showing their technical skills, taqsīm allows the players to interact as active agents.

### Interaction

Hill (2018) points out that the next component of her cross-cultural model of musical creativity can be shadowed – especially in western culture – by agency, namely the interaction component. According to Hill (2018, p. 5) “(i)nteraction includes being stimulated by and responding to input from musicians, audience members, and the environment.” Interaction, according to Hill, includes being engaged with the other musicians with whom one plays and with the audience. During taqsīm performance, the same musicians may perform in varied styles, depending largely upon their emotional state during the performance and upon the nature of their audience (Racy, 1991).

Our participants expressed that interaction is an important aspect of taqsīm. For Ala’a, taqsīm “will change because there are other people [an audience].” Moreover, having other players with whom one plays changes taqsīm as well because there is “competition with the other players.”

For Nabil, taqsīm is “a direct interaction between the player and the audience.” To Nabil, such interaction is multidimensional “[with] the audience on one hand and [with] the band [with whom one plays] on the other hand.”

For Wassim, taqsīm is “meant to highlight the technical skills of the player, his understanding of his instrument and interaction with the audience and with the music itself and also with other players.”

In addition to the interaction with the audience and with other musicians through taqsīm, as expressed by Ala’a and Nabil, Wassim added that taqsīm is in interaction with the (pre-composed) music itself, especially when taqsīm is played before or within a piece:

“In my opinion, the taqsīm has essentially two roles: the first role is to introduce the pieces by bringing the musicians and the audience into a state where there is a preparation for the atmosphere of the piece. Later to get into the piece, so that the piece does not come as a surprise […]. This is the first role. The second role is to have one or more solos for one or more instruments within the piece before the conclusion […].”

The participants’ answers point out that interaction while performing taqsīm might manifest itself in various ways and on various levels. There is interaction with the audience, other musicians, and with the music itself.

### Non-conformity

To encompass a cluster of concepts such as novelty, innovation, difference, and originality, Hill (2018) uses the term non-conformity, as the fourth component of her model of musical creativity. Hill argues that this term helps to (a) explain why and how creativity is restricted by social pressures to conform to norms and standards and (b) to note that innovation and novelty may not always be valued within music cultures that prioritize historical authenticity and preservation of tradition. However, Hill points out that this does not mean that such traditionally oriented music cultures do not allow for creativity. On the contrary, according to her, an extensive expression of creativity can exist within the bounds of tradition. Hill notes that the creative musician explores and realizes multiple possibilities instead of conforming to an entirely predetermined model. According to Ayari and McAdams (2003), taqsīm is organized into several phases in the presentation and development of each maqam. However, they note that the precise ordering is not fixed: introduction, presentation of the basic maqam elements, exposition, re-exposition, and confirmation. Ayari and McAdams add that some performers may linger on a given phase of taqsīm before moving to the execution of another idea. They add that the duration of each phase depends on the artistic mastery of each performer. Such a flexible form may facilitate non-conformity.

Our participants expressed their attempt to integrate musical material from sources other than Arabic musical tradition. For example, Ala’a borrows “some techniques of Turkish music” and “a combination of jazz and blues, especially after coming to Europe and learning more about these styles and playing with bands that play these styles.” More specifically, Ala’a noted he has “a tendency to use certain blues scales in the bridge of the maqam while keeping the shape of the maqam.”

For Nabil, being in Berlin gave him the chance to encounter electronic music. “I am trying to learn the technique of repetition in electronic music.” He described how he can deviate from traditional musical ideas:

“I have moved away from the basic musical ideas and the main melodies of the [original] musicians. I created melodies that I like, and decided to concentrate on the ideas that I wanted to create to build a taqsīm that would convince me.”

Wassim integrated elements of Azerbaijani music into his taqsīm style because one of his teachers was Azerbaijani. Moreover, moving to Berlin and having a new audience gave him more freedom to vary his taqsīm. This freedom comes from the fact that his audience in Berlin includes – in addition to listeners familiar with Arabic music – listeners familiar with Turkish music or European classical music. Thus he has the freedom to vary his taqsīm and he does not have to stick with only the “Arabic way” of playing taqsīm.

These insights show that the participants were seeking to integrate non-traditional elements into their own taqsīm. These elements are related to their interest in other musical genres and styles. Moreover, there is some intersection in their preferences and choices. However, the elements they choose to integrate into their taqsīm may vary considerably: they could be musical phrases (building blocks), technical skills, or esthetic principles.

### Recycling

The fifth component of Hill’s model of musical creativity is recycling. Hill (2018) notes that creative processes also depend on recycling, or reusing, remixing, and otherwise building on, tradition. Racy (2000) notes that a taqsīm in a specific maqam, or melodic mode, tends to be self-contained. This means that it is begun, developed, and resolved in accordance with an established modal plan. In many cases, the material that builds such a structure is borrowed. Watson (2012) points out that, even though most taqsīm performers view it as having no rules as such, there is certainly a necessity for traditional musical details to be enacted during the performance. Such details, Watson notes, are internalized by modeling musical behavior on traditional prototypes. Watson adds that the methods through which musicians develop the ability to produce genre-specific musical elements are enabled by engaging in various learning environments such as parental apprenticeships, informal apprenticeships with master musicians, vocalists and ensembles, musician networks, autodidactic study, and institutionalized study. Watson (2012) points out that musicians who play taqsīm and other genres absorb pre-composed melodic prototypes as stylistic and theoretical blueprints.

Ala’a stated that “borrowing phrases from a song or an older work is a normal thing, because this is the repertoire of the musician that gets realized and embodied.”

Nabil describes listening to taqsīm masters and trying “to repeat them as if they were pieces.” Moreover, Nabil’s teachers told him that “the musician gets richer every time he learns pieces and taqsīms from other musicians.” Nabil elaborated: “Sometimes it is possible to build a taqsīm from a song without the audience noticing. The musician can take ideas from a song and change them—or from a melody or a piece—and make a taqsīm out of it.”

For Wassim, the melodies heard within taqsīm have different sources, but mainly they are based on “sequences of the tetrachords and on the repertoire of each musician.”

These insights from our interviews show that it is a common practice to use recycled materials when playing taqsīm. These materials may be motives or melody segments from songs and other pieces or more abstract building blocks like maqams.

### Flow

Flow is the sixth component of Hill’s model of musical creativity (Hill, 2018). She points out that Csikszentmihalyi, (1996, p. 110) defines flow as “the feeling when things were going well as an almost automatic, effortless, yet highly focused state of consciousness.” According to Shannon (2003), music lovers and performers of the classical Arab musical repertoire associate esthetic quality and authenticity with the ability of artists and their audiences to achieve tarab. Shannon (2003, p. 74) further notes that in Arabic, tarab refers linguistically to “a state of heightened emotionality, often translated as ‘rapture,’ ‘ecstasy,’ or ‘enchantment’ but can also indicate sadness as well as joy.” Shannon adds that tarab also describes a style of music and musical performance which evoke such emotional states in performers and audiences. According to Shannon (2003), in the performance of a taqsīm, an artist can establish a sense of *saltana* which Shannon describes as melodic flow or groove. Shannon suggests that these strategies have the effect of altering the listener’s experience of temporality. According to Shannon, the experience of detemporalization and retemporalization may in fact be critical to the production of tarab. Shannon points out that although technical ability may generate excitement among audiences, for listeners, the artists’ ability to alter the experience of time is a primary indicator of their creativity and authenticity. Shannon notes that listening to a traditional taqsīm brings listeners out of the normal flow of time where melodic repetition and fluency of movement create a sense of suspended time.

In our study, the musicians expressed comparable opinions. Ala’a stated that “when the time of the taqsīm comes and I play my taqsīm, I may close my eyes because I am building inner images and experiencing a certain situation.” Ala’a expressed a common experience related to flow, which is forgetting the surroundings.

Nabil feels “absolutely free in solo concerts.” Moreover, he describes how he plays his music for himself: “A large part of the music is not for other people. I do what I am convinced of, what I feel.” Following his feelings without a pre-established plan can be seen as an important part of the flow experiences.

When Wassim plays on his own he feels that he is free and able to “enter into a situation that is similar to a dialogue with the self and [similar to] a Sufi experience.” He added that he may enter a state comparable to flow when playing on his own, where he does not feel the pressure of having an audience: “I don’t have the crisis [the issue, the question] whether the audience is enjoying it or not. Therefore, I can take long breaks and enter into a situation that is closer to the dialogue with the self and a Sufi process… Meditation through the music.” When asked about playing with others, Wassim stated:

“The solo player [in such a situation] has less freedom for the taqsīm, but at the same time there is a higher level of communication, because at that moment [the whole] ensemble can participate [in playing, and this can be] a spiritual experience.”

**FIGURE 1:**
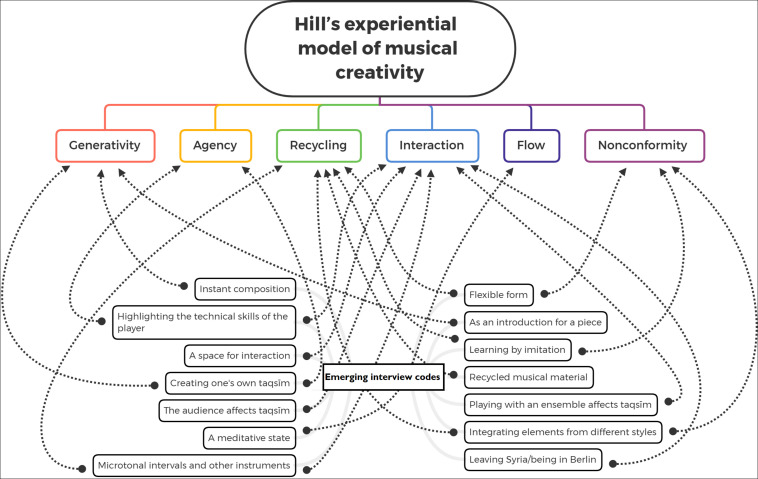
Mapping between the interview codes and Hill’s model components of musical creativity (generativity, agency, interaction, non-conformity, recycling, and flow). All components of Hill’s model were evident in our analysis of the particpants’ answers on taqsīm.

Wassim’s statement may point to a sense of *saltana* which Shannon (2003) describes as melodic flow or groove. Yet these experiences need further investigation to find out whether *saltana* is part of a flow experience or not. The mapping of the interview final codes and Hill’s model is shown in Figure 1.

### Tensions

Hill (2018) points out that all these components—generativity, agency, interaction, non-conformity, recycling, and flow—usually coexist and overlap. However, tensions between them may occur. According to Hill, interaction and recycling can be hindered by too much focus on individual agency and non-conformity. Moreover, agency, non-conformity, and flow can be hindered by too much concern about external evaluation.

We found some indications of tensions in our participants’ answers. These tensions might be a part of interaction in different situations. For example, Ala’a noted the audience evaluation is important during taqsīm:

“Maybe the musician experiences an ideal state, that is, he feels that he is playing a beautiful taqsīm, but the audience is wondering what this person is doing on stage.”

This statement shows that there might be a tension between agency and external evaluation of the creative process. Nabil pointed out that tensions may occur among musicians playing together. He might wish to play a free taqsīm without a pre-determined duration but this can be irritating to other musicians:

“A long taqsīm puts pressure on the other musicians on stage, who are waiting for it to end!”

Moreover, he noted that taqsīm—when performed within a piece—should fit that piece, which may create a tension between the generated material on stage and the pre-composed piece:

“I can think that the taqsīm is beautiful and the audience enjoys it, but it doesn’t fit well with the piece.”

In addition to that, playing with an orchestra may limit the freedom of taqsīm:

“For example, they [the orchestra, other musicians] asked me to create a clear and specific duration of taqsīm. So I had to make a taqsīm that is similar to a composition, because I had to know exactly what I was going to do and play.”

These examples given by Nabil point out a pressure to adapt to other musicians, pre-composed pieces, or to other musical practices (like playing with an orchestra). A further tension may take place between the desire to be creative and the concern about feedback from the audience. Nabil stated:

“I try to play original material in every concert, when I do so, I feel happy and comfortable and feel that the taqsīm is more than just a repetition. […] When I play for an audience, there is a lack of oxygen in my body because I am nervous. Also, because I’m afraid of making mistakes. I have to say that [there] I am less brave. My [musical] thoughts at home are always more important, because even if I make a mistake, big or small, I can repeat and repeat the phrase [phrases] until I play it correctly. On stage, that’s not allowed, I play the safest phrases on stage while I’m free on my own.”

This tension, which may limit non-conformity, results from the concern about external evaluation. Wassim noted a similar tension when playing with others: “the solo player [in such a situation] has less freedom for the taqsīm.”

Moreover, when talking about taqsīm’s functions, Wassim noted that tension may occur between the wish to play virtuously and the introductory function of taqsīm:

“This second function is to highlight the player’s skills, while the first function is to support and prepare for the music. Preferably, the player does not play very virtuously [in the first function], so as not to divert full attention from the piece.”

This can be described as a tension between recycling and individual agency.

Moreover, Wassim stated:

“When I play alone, I generally don’t have to give a pattern or explain my style, so I don’t have to do the same repetitions and I can do fewer repetitions. At the same time, I don’t have the crisis [the issue, the question] whether the audience is enjoying it or not. Therefore, I can take long breaks and enter into a situation that is closer to the dialogue with the self and a Sufi process… Meditation through the music – I try to keep that away from the stage, especially at concerts when there is a mixed audience of different cultures. I try to make the taqsīm clear and understandable for the wider audience. This is because I will lose a part of the audience if I radicalize my music in any direction [being too meditative, or making it so clear]. But when I am alone, I can go in the direction I want and without losing anything.”

Wassim’s statement shows that he might experience a tension between the wish of entering a meditative state, where his music cannot be completely understood or followed by his listeners, and trying to keep his music clear and concentrated at the price of not entering such a meditative state. This can be described as a tension between agency, non-conformity, and flow on the one hand and with the concern about external evaluation on the other. The participants’ answers show that tension may appear between many different components: between recycling and novelty, freedom and limitation, the artistic expression of the moment and the coherence of the concert program etc.

## Conclusion

The aim of this study was to shed light on taqsīm as a creative process from a transcultural point of view. The interviews with the three Berlin-based oud players from Syria revealed that taqsīm is a kind of instant composition with a flexible form that highlights the technical skills of the player and/or serves as an introduction for pre-composed music. Moreover, it serves as a space for interaction between the musicians, the audience and the pre-composed music. Taqsīm is learned by imitation and experimenting, and contains recycled musical materials from maqam music amongst others. The migratory situation enabled our participants to have new musical experiences and to integrate new musical materials, playing techniques and esthetic values into their taqsīm. Their contact with various musical genres in Berlin—in addition to experiencing diverse concert audiences—gave our participants new opportunities to negotiate the boundaries of taqsīm. We mapped the codes and themes of the thematic content analysis onto Hill’s cross-cultural experiential model of musical creativity. This mapping showed that: (1) generativity in taqsīm is goal-oriented. Generating taqsīm aims to create room for individual artistic expression, for interaction with the audience, and to support and introduce the pre-composed pieces. (2) Due to the freedom of taqsīm, and due to its role in showing the technical skills of the musicians, taqsīm allows the players to interact as active agents. (3) There is interaction with the audience, other musicians, and with the music itself. (4) The participants were seeking to integrate non-traditional elements in their own taqsīm. (5) It is a common practice to use recycled materials when playing taqsīm. (6) A state of flow or meditative experience when one forgets the surrounding may appear when playing taqsīm. Some of the tensions that may appear between the different components of Hill’s model were evident, too, in our analysis of the interviews. Such tensions may appear between recycling and novelty, freedom and limitation, the artistic expression of the moment and the coherence of the concert program.

Hill’s model of creativity—which highlights the components of generativity, agency, interaction, non-conformity, recycling, and flow—may facilitate positioning, linking, and comparing taqsīm with other musical genres and cultures. Such a theoretical framework provides the opportunity to investigate general features that characterize many creative musical practices without denying the specific details of each genre. The multidimensional view of taqsīm as a creative practice with multiple components appears to be more fruitful in capturing the full breadth of this musical practice than placing it on an improvisation-composition continuum. We envisage that having applied Hill’s model in this study will further open up pathways for incorporating transcultural musical processes in the study of creativity.

## Data Availability Statement

The raw data supporting the conclusions of this article will be made available by the authors, without undue reservation.

## Ethics Statement

Ethical review and approval was not required for the study on human participants in accordance with the local legislation and institutional requirements. The participants provided their written informed consent to participate in this study. Written informed consent was obtained from the individual(s) for the publication of any potentially identifiable images or data included in this article.

## Author Contributions

ZA and MBK developed the idea and design of the study. ZA conducted the interviews, analyzed the data, and produced the first draft. MBK provided substantial comments and assisted with the argument development. Both authors approved the final version of the manuscript.

## Conflict of Interest

The authors declare that the research was conducted in the absence of any commercial or financial relationships that could be construed as a potential conflict of interest.

## Footnotes

1 Hill uses the term experiential to emphasize the musicians’ experience which distinguishes her research from the common creativity literature, which focuses on external evaluation of creative products, J. Hill (personal communication, March 15, 2021).
